# Supplementary material for: Economic events and the volatility of government bill rates
Source: PLoS One. 2022 Oct 19;17(10):e0276345. doi: 10.1371/journal.pone.0276345 (PMC9581437; doi:10.1371/journal.pone.0276345)
Supplement: S1 Appendix — (DOCX) [file pone.0276345.s002.docx]

**Appendix A**

**Proof of Proposition 1.** The logarithm of Eq. (1) is $\ln U_{t}=\frac{\ln\left\{ \left( 1-\delta\right)\left( C_{t} \right)^{1-1/\psi}+\delta E_{t}\left[ \left( U_{t+1} \right)^{1-\gamma} \right]^{\frac{1-1/\psi}{1-\gamma}} \right\}}{1-1/\psi}$. Using L'Hospital's rule, the limit of it when $\psi=1$ is $\lim_{\psi\to1} \frac{\ln\left\{ \left( 1-\delta\right)\left( C_{t} \right)^{1-1/\psi}+\delta E_{t}\left[ \left( U_{t+1} \right)^{1-\gamma} \right]^{\frac{1-1/\psi}{1-\gamma}} \right\}}{1-1/\psi}=\left( 1-\delta\right)\ln C_{t}+\frac{\delta}{1-\gamma}\ln E_{t}\left[ e^{\left( 1-\gamma\right)\ln U_{t+1}} \right]$. This completes the proof.

**Appendix B**

**Proof of Proposition 2.** Since all correlations are zero according to assumption 1,

$E_{t}\left( e^{\left( 1-\gamma\right)\zeta_{t+1}} \right)=E_{t}\left[ e^{\left( 1-\gamma\right)\left( \varpi_{t+1}+\omega_{1}N_{1,t+1}+\omega_{2}N_{2,t+1} \right)} \right]=E_{t}\left[ e^{\left( 1-\gamma\right)\varpi_{t+1}} \right]E_{t}\left[ e^{\left( 1-\gamma\right)\omega_{1}N_{1,t+1}} \right]E_{t}\left[ e^{\left( 1-\gamma\right)\omega_{2}N_{2,t+1}} \right].$ (B1)

According to assumption 1, $E_{t}\left[ e^{\left( 1-\gamma\right)\varpi_{t+1}} \right]=e^{\left( 1-\gamma\right)v_{t}+\frac{\left( 1-\gamma\right)^{2}}{2}\chi_{t}^{2}}$. $N_{i,t+1}$ follows Bernoulli distribution as shown in Eq. (5), so $E_{t}\left[ e^{\left( 1-\gamma\right)\omega_{i}N_{i,t+1}} \right]=\lambda_{i,t}e^{\left( 1-\gamma\right)\omega_{i}}+1-\lambda_{i,t}.$ $i\in\{1,2\}$. This completes the proof.

**Appendix C**

**Proof of Proposition 3.** Eq. (2) can be rewritten as $\ln U_{t}-\ln C_{t}=\frac{\delta}{1-\gamma}\ln E_{t}\left[ e^{\left( 1-\gamma\right)\ln U_{t+1}} \right]-\delta\ln C_{t}=\frac{\delta}{1-\gamma}\ln E_{t}[e^{\left( 1-\gamma\right)\left( \ln U_{t+1}-\ln C_{t} \right)}]$, where $y_{t}=\ln U_{t}-\ln C_{t}$ and $\zeta_{t+1}=\ln U_{t+1}-\ln C_{t}$. Based on Eq. (10), it’s easy to get Eq. (11). This completes the proof.

**Appendix D**

**Proof of Proposition 4.** $\zeta_{t+1}=y_{t+1}+\Delta c_{t+1}$. Using assumption 3, $\zeta_{t+1}=l_{1}+\mu+l_{4}\theta_{1}+l_{5}\theta_{2}+\left( 1+l_{2} \right)a_{1}\eta_{1,t}+\left( 1+l_{3} \right)a_{2}\eta_{2,t}+l_{4}\phi\lambda_{1,t}+l_{5}\phi\lambda_{2,t}+\sigma\varepsilon_{t+1}+l_{4}\sigma_{\lambda}\sqrt{\lambda_{1,t}}\varepsilon_{1,t+1}+l_{5}\sigma_{\lambda}\sqrt{\lambda_{2,t}}\varepsilon_{2,t+1}+\left( 1+l_{2} \right)b_{1}N_{1,t+1}+\left( 1+l_{3} \right)b_{2}N_{2,t+1}$. Thus, assumption 1 shows that $v_{t}=l_{1}+\mu+l_{4}\theta_{1}+l_{5}\theta_{2}+\left( 1+l_{2} \right)a_{1}\eta_{1,t}+\left( 1+l_{3} \right)a_{2}\eta_{2,t}+l_{4}\phi\lambda_{1,t}+l_{5}\phi\lambda_{2,t}$, $\chi_{t}^{2}=\sigma^{2}+\left( l_{4}\sigma_{\lambda} \right)^{2}\lambda_{1,t}+\left( l_{5}\sigma_{\lambda} \right)^{2}\lambda_{2,t}$, $\omega_{1}=\left( 1+l_{2} \right)b_{1}$ and $w_{2}=\left( 1+l_{3} \right)b_{2}$. Eq. (11) implies that $y_{t}=\delta\left[ l_{1}+\mu+l_{4}\theta_{1}+l_{5}\theta_{2}+\left( 1+l_{2} \right)a_{1}\eta_{1,t}+\left( 1+l_{3} \right)a_{2}\eta_{2,t}+l_{4}\phi\lambda_{1,t}+l_{5}\phi\lambda_{2,t} \right]+\frac{\delta\left( 1-\gamma\right)}{2}\left[ \sigma^{2}+\left( l_{4}\sigma_{\lambda} \right)^{2}\lambda_{1,t}+\left( l_{5}\sigma_{\lambda} \right)^{2}\lambda_{2,t} \right]+\delta\left( 1+l_{2} \right)b_{1}\lambda_{1,t}+\delta\left( 1+l_{3} \right)b_{2}\lambda_{2,t}$. Using assumption 3 again,

$l_{1}=\delta\left[ l_{1}+\mu+l_{4}\theta_{1}+l_{5}\theta_{2} \right]+\delta\sigma^{2}\left( 1-\gamma\right)/2,$ (D1)

$l_{2}=\delta\left( 1+l_{2} \right)a_{1},$ (D2)

$l_{3}=\delta\left( 1+l_{3} \right)a_{2},$ (D3)

$0=\left( \frac{1}{2} \right)\delta\left( 1-\gamma\right)\sigma_{\lambda}^{2}l_{4}^{2}+\left( \delta\phi-1 \right)l_{4}+\delta\left( 1+l_{2} \right)b_{1},$ (D4)

$0=(1/2)\delta\left( 1-\gamma\right)\sigma_{\lambda}^{2}l_{5}^{2}+\left( \delta\phi-1 \right)l_{5}+\delta\left( 1+l_{3} \right)b_{2}$. (D5)

From Eqs. (D1), (D2), and (D3), it is easy to calculate $l_{1}$, $l_{2}$ and $l_{3}$: $l_{2}=\frac{\delta a_{1}}{1-\delta a_{1}}$, $l_{3}=\frac{\delta a_{2}}{1-\delta a_{2}}$ and $l_{1}=\frac{\delta\left( \mu+l_{4}\theta_{1}+l_{5}\theta_{2} \right)+\delta\sigma^{2}\left( 1-\gamma\right)/2}{1-\delta}$. Both Eqs. (D4) and (D5) have two roots. If we take the smaller root of Eqs. (D4) and (D5), $\lim_{\sigma_{\lambda}\to0} l_{4}=-\infty$ and $\lim_{\sigma_{\lambda\to0}} l_{5}=-\infty$. Thus, only the larger root of Eqs. (D4) and (D5) makes economic sense: $l_{4}=\frac{1-\delta\phi-\sqrt{\left( 1-\delta\phi\right)^{2}-2\left( 1-\gamma\right)\delta^{2}\sigma_{\lambda}^{2}\left( 1+l_{2} \right)b_{1}}}{\delta\left( 1-\gamma\right)\sigma_{\lambda}^{2}}$ and $l_{5}=\frac{1-\delta\phi-\sqrt{\left( 1-\delta\phi\right)^{2}-2\left( 1-\gamma\right)\delta^{2}\sigma_{\lambda}^{2}\left( 1+l_{3} \right)b_{2}}}{\delta\left( 1-\gamma\right)\sigma_{\lambda}^{2}}$. This completes the proof.

**Appendix E**

**Proof of Proposition 5.** Solving the Euler equation $\left( E_{t}\left[ e^{m_{t+1}+r_{t+1}^{c}} \right]=1 \right)$ based on assumptions 5 and 6 can yield the following equations:

$0=\ln\left[ \delta\left( e^{pc}+1 \right) \right]\mathcal{-l}pc+\left( \mathcal{l-}1 \right)A_{1}^{c}\mathcal{+l}\left( A_{4}^{c}\theta_{1}+A_{5}^{c}\theta_{2} \right),$ (E1)

$0=\mathcal{l}A_{2}^{c}a_{1}-A_{2}^{c},$ (E2)

$0=\mathcal{l}A_{3}^{c}a_{2}-A_{3}^{c},$ (E3)

$0=\left( 1/2 \right)\mathcal{l}^{2}\delta^{2}\left( A_{4}^{c} \right)^{2}-\left[ 1-\mathcal{l}\phi\mathcal{-l}\left( 1-\gamma\right)l_{4}\sigma_{\lambda}^{2} \right]A_{4}^{c}\mathcal{+l}A_{2}^{c}b_{1},$ (E4)

$0=\left( 1/2 \right)\mathcal{l}^{2}\delta_{\lambda}^{2}\left( A_{5}^{c} \right)^{2}-\left[ 1-\mathcal{l}\phi\mathcal{-l}\left( 1-\gamma\right)l_{5}\sigma_{\lambda}^{2} \right]A_{5}^{c}\mathcal{+l}A_{3}^{c}b_{2},$ (E5)

From Eqs. (E2) and (E3), it is easy to calculate: $A_{2}^{c}=0$, $A_{3}^{c}=0$. Both Eqs. (E4) and (E5) have two roots. If we take the larger root of Eqs. (E4) and (E5), $\lim_{\sigma_{\lambda\to0}} A_{4}^{c}=\infty$ and $\lim_{\sigma_{\lambda\to0}} A_{5}^{c}=\infty$. Thus, only the smaller root of Eqs. (E4) and (E5) makes economic sense: $A_{4}^{c}=A_{5}^{c}=0$. So, from Eq. (E1), $A_{1}^{c}=\frac{\ln\left[ \delta\left( e^{pc}+1 \right) \right]\mathcal{-l}pc}{1-\mathcal{l}}$. This completes the proof.
