# Supplementary material for: Economic events and the volatility of government bill rates
Source: PLoS One. 2022 Oct 19;17(10):e0276345. doi: 10.1371/journal.pone.0276345 (PMC9581437; doi:10.1371/journal.pone.0276345)
Supplement: Attachment — Submitted filename: Response to Reviewers.docx [file pone.0276345.s003.docx]

Dear Reviewers:

Thank you for the constructive comments concerning our manuscript. Those comments are all valuable and very helpful for revising and improving our paper. We have studied your comments carefully and have made revisions which we hope meet with your approval. In the following, we provide point‐by‐point responses to the comments raised by the reviewers (see below for details). All the changes in the revised manuscript are marked as red characters.

It is important to clarify that while we present the original manuscript in PDF format, we present the revised manuscript in WORD format. Our revisions are based on the original manuscript in WORD format. This is mainly to show you the revision process more clearly and to satisfy common review habits. We promise that the original manuscript in WORD format has exactly the same content as the original manuscript in PDF format that we have already submitted. Therefore, each of your comments applies to our original manuscript in WORD format.

We thank you again for your attentiveness and professionalism before responding formally.

**Response to Reviewer 1's Comments**

**Point 1:** Section simulation: A simulation study is provided to demonstrate the proposed methodology. Clearly discuss the choices of the stated models and the parameters used.

**Response 1:** Thank you very much! We have rewritten the section “Simulation” of the original manuscript.

**Point 2:** The authors have provided some propositions and each the proof is provided. I would suggest the authors include those important proofs in the appendix such as proposition 1.

**Response 2:** Thank you very much! Inspired by your suggestion, we see the benefits of putting all the proofs in the appendix section: i) it makes the structure of the manuscript clearer; ii) it makes the manuscript more accessible; iii) it makes the main results of the manuscript more prominent. We have placed all the proofs in the appendix of the revised manuscript. Following your suggestion, we feel that the readability and aesthetics of the manuscript have been improved in general.

**Point 3:** Figure 3: (i) What is the value of b? (ii) Explain the claim “Lines 151-152: it is not a very good fit to the distribution of transformed sizes of the series” and provide statistical evidence to justify your claims. (iii) label the x- and y-axes. (iv) How to obtain the equations $y=40\times z^{-30}$ and $y=15\times z^{-20}$.

**Response 3:** Thank you very much!

(i) $b$ represents the size of the rises or declines. For example, if a trough-to-peak absolute rise is on a size of 10%, $b$ is equal to 0.1; if a peak-to-trough absolute decline is on a size of 5%, $b$ is equal to 0.05.

(ii) This sentence is saying that the distribution of transformed sizes of the rises and declines defined by us cannot be well fitted by a power-law density. To confirm this claim, we redraw “Figure 3” with a smaller width of the bin (see Figure 1 in this document). If the distribution of transformed sizes can be well fitted by a power-law density, the frequency will generally increase as the transformed size decreases. However, such a change trend is not obvious in Figure 1.

**Figure 1. The distribution of transformed sizes of the rises and declines in**

**U.S. consumption growth rate data.**

(iii) “Figure 3” can be confusing: the distribution of transformed sizes seems to be well fitted by a power-law density. As shown in Figure 1, this is actually not the case. Therefore, in the revised manuscript, we intend to replace "Figure 3" with Figure 1. In the original manuscript, we give equations $y=40\times z^{-30}$ and $y=15\times z^{-20}$ roughly to show that the distribution of transformed sizes cannot be well fitted by a power-law density, where $z$ and $y$ represent the transformed size and frequency, respectively. We no longer plot any equations in Figure 1 because the distribution of transformed sizes in Figure 1 is clearly not well fitted by a power-law density.

**Point 4:** Line 159: Figure 5 provides different plots and not descriptive statistics. Explain what is meant by “Figure 5 shows descriptive statistics of events with different event thresholds.”

**Response 4:** Thank you very much for pointing out this error. We have realized that it would be inappropriate to title Figure 5 as "descriptive statistics of events at different event thresholds". In fact, we intend to show the correspondence between the average duration and thresholds, the correspondence between the average cumulative effect and thresholds, and the correspondence between the probability and thresholds in Figure 5. Therefore, as shown in Table 1, we rewrite the title of Figure 5.

**Table 1 Correct title errors in the original manuscript**

|  | *Original manuscript in PDF format* | | *Revised manuscript in WORD format* | |
| --- | --- | --- | --- | --- |
|  | Line(s) |  | Line(s) |  |
| 1 | 159-160 | Fig 5 shows descriptive statistics of events with different event thresholds. | 241-242 | Fig 5 shows the correspondence between thresholds and characteristics of economic events. |
| 2 | 169-170 | **Fig 5. Descriptive statistics of economic events under different thresholds.** | 252-253 | **Fig 5. Thresholds and characteristics of economic events.** |

**Point 5:** There are some typos and spelling errors to be corrected such as line 49: “Dad”, line 111: “Our model”. The authors should rewrite and check the paper thoroughly.

**Response 5:** Thank you very much! We thoroughly checked the manuscript for typos and spelling errors and corrected them all. As shown in Table 2, we correct 22 errors in total.

**Table 2.** **Correct typos and spelling errors in the original manuscript**

| *Original manuscript in PDF format* | | *Revised manuscript in WORD format* | |
| --- | --- | --- | --- |
| Line(s) | Typos and spelling errors | Line(s) | Correct typing and spelling |
| 27 | indicates | 95 | indicate |
| 30 | Barro and Jin [13] | 97 | Barro and Jin [15] |
| 49 | Dad | 116 | Bad |
| 94 | for | 167 | of |
| 95 | our model | 167 | Our model |
| 111 | our model | 184 | Our model |
| 132 | the disaster probability | 207 | disaster probability |
| 171-172 | $U_{t}=\left[ \left( 1-\delta\right)\left( C_{t} \right)^{1-\psi}+\delta E_{t}\left[ \left( U_{t+1} \right)^{1-\gamma} \right]^{\frac{1-\psi}{1-\gamma}} \right]^{\frac{1}{1-\psi}}$ | 256 | $U_{t}=\left[ \left( 1-\delta\right)\left( C_{t} \right)^{1-1/\psi}+\delta E_{t}\left[ \left( U_{t+1} \right)^{1-\gamma} \right]^{\frac{1-1/\psi}{1-\gamma}} \right]^{\frac{1}{1-1/\psi}},$ |
| 183 | that, | 267 | that |
| 189 | $\ln U_{t}=\frac{\ln\left\{ \left( 1-\delta\right)\left( C_{t} \right)^{1-\psi}+\delta E_{t}\left[ \left( U_{t+1} \right)^{1-\gamma} \right]^{\frac{1-\psi}{1-\gamma}} \right\}}{1-\psi}$ | 720 | $\ln U_{t}=\frac{\ln\left\{ \left( 1-\delta\right)\left( C_{t} \right)^{1-1/\psi}+\delta E_{t}\left[ \left( U_{t+1} \right)^{1-\gamma} \right]^{\frac{1-1/\psi}{1-\gamma}} \right\}}{1-1/\psi}$ |
| 191 | $\frac{\ln\left\{ \left( 1-\delta\right)\left( C_{t} \right)^{1-\psi}+\delta E_{t}\left[ \left( U_{t+1} \right)^{1-\gamma} \right]^{\frac{1-\psi}{1-\gamma}} \right\}}{1-\psi}$ | 721 | $\frac{\ln\left\{ \left( 1-\delta\right)\left( C_{t} \right)^{1-1/\psi}+\delta E_{t}\left[ \left( U_{t+1} \right)^{1-\gamma} \right]^{\frac{1-1/\psi}{1-\gamma}} \right\}}{1-1/\psi}$ |
| 196 | comsuption | 282 | consumption |
| 199 | the short-term shock | 285 | the short-run shock |
| 199 | comsuptions | 285 | consumption |
| 204 | long-run risk mode | 291 | the long-run risk mode |
| 209 | (3), (4) and (5) | 297 | (3), (4), and (5) |
| 264 | and | 368 | , and |
| 316 | $r_{t+1}^{d}$ | 444 | $r_{t+1}^{e}$ |
| 325-326 | **Fig 9. Equity return volatility under different expected event probabilities.** Set $\Phi=3$, $\sigma=0.02$, $\sigma_{d}=0.15$, $\phi=0.92$, $\sigma_{\lambda}=0.1$, $a_{1}=a_{2}=0.15$ and $b_{1}={-b}_{2}=0.1$. Let $E\left( \lambda_{1.t} \right)=E\left( \lambda_{2.t} \right)=E\left( \lambda_{t} \right)$ when both good and bad events are modeled. Let $E\left( \lambda_{1.t} \right)=0$ and $E\left( \lambda_{2,t} \right)=E\left( \lambda_{t} \right)$ when only bad events is modeled. | 456 | **Fig 9. Equity return volatility with different values of the event probability.** |
| 325-326 | **Fig 10. Equity return volatility under different volatility parameters.** Set $\Phi=3$, $\sigma=0.02$, $\sigma_{d}=0.15$, $\phi=0.92$, $\sigma_{\lambda}=0.1$, $a_{1}=a_{2}=0.15$ and $b_{1}=-b_{2}=0.1$. Let $E\left( \lambda_{1.t} \right)=E\left( \lambda_{2.t} \right)=0.1$ when both good and bad events are modeled. Let $E\left( \lambda_{1.t} \right)=0$ and $E\left( \lambda_{2,t} \right)=0.1$ when only bad events is modeled. | 460 | **Fig 10. Equity return volatility with different values of the volatility parameter.** |
| 363-364 | $\Upsilon_{1}$ | 548-549 | $\Upsilon_{2}$ |
| 373-374 | $\Upsilon_{1}$ | 597-598 | $\Upsilon_{2}$ |

**Point 6:** Line 30: “Barron and Jin [13]”. Incorrect citation.

**Response 6:** Thank you very much! We have corrected “Barron and Jin [13]” to “Barron and Jin [15]” in the revised manuscript.

**Point 7:** Abstract: Rephase some sentences such as “This paper extended…” and “In this paper, …”.

**Response 7:** Thank you very much! We carefully checked every sentence in the abstract and rephrased some sentences. The abstract in the revised manuscript is as follows:

“Many studies show that in many countries (especially the G7), volatility in government bill rates far exceeds that in consumption growth rates. This volatility puzzle cannot be predicted by traditional disaster models, in which rare economic disasters are defined as a peak-to-trough percent fall in consumption (or real per capita GDP) by a high threshold ($\geq10\%$). For this purpose, we extend the traditional definition of rare economic disasters and propose a novel asset pricing model that models both good and bad events. We define a bad (or good) event as a peak-to-trough absolute decline (or a trough-to-peak absolute rise) in consumption growth rates by a low threshold ($<10\%$). Compared to traditional disaster models, our model contains three improvements. First, model good and bad events, not just bad ones (e.g., rare economic disasters). Second, the event’s impact lasts for multiple periods rather than one period. Third, model non-rare economic events. We calibrate the parameters in our model to match the moments from U.S. asset return data. Simulation results indicate that the model can successfully predict the volatility of U.S. government bill rates higher than that of U.S. consumption growth rates.”

**Point 8:** Line 198: Replace “.” with “,” after “where delta_c, mu, epsilon and etc”. Similar comment applied for other section.

**Response 8:** Thank you very much! As shown in Table 3, we corrected all similar errors in the original manuscript.

**Table 3. Correct some punctuation errors in the original manuscript**

| *Original manuscript in PDF format* | *Revised manuscript in WORD format* |
| --- | --- |
| Line(s) | |
| 172-174 | 257-259 |
| 198-199 | 284-285 |
| 213-214 | 301-302 |
| 221-223 | 311-313 |
| 293-295 | 409-411 |

**Point 9:** Some papers in the list of references with incomplete information and incorrect formatting.

**Response 9:** Thank you very much! As shown in Table 4, we have added information to some references and corrected the formatting of some references.

**Table 4. Correct some reference errors in the original manuscript**

*Original manuscript in PDF format:*

4.Barro RJ, Ursúa JF. Macroeconomic crises since 1870. National Bureau of Economic Research. 2008. <https://doi.org/10.3386/w13940>.

*Revised manuscript in WORD format:*

4.Barro RJ, Ursúa JF. Macroeconomic crises since 1870. National Bureau of Economic Research. 2008. <https://doi.org/10.3386/w13940>.

*Original manuscript in PDF format:*

7.Wachter JA. Can the time-varying risk of rare disasters explain aggregate stock market volatility?. The Journal of Finance. 2013; 68(3): 987-1035. <https://doi.org/10.3386/w14386>.

*Revised manuscript in WORD format:*

7.Wachter JA. Can the time-varying risk of rare disasters explain aggregate stock market volatility? The Journal of Finance. 2013; 68(3): 987-1035. <https://doi.org/10.3386/w14386>.

*Original manuscript in PDF format:*

10.Julliard C, Anisha G. Can rare events explain the equity premium puzzle?. Review of Financial Studies. 2012; 25 (10): 3037-76. <https://doi.org/10.1093/rfs/hhs078>.

*Revised manuscript in WORD format:*

10.Julliard C, Anisha G. Can rare events explain the equity premium puzzle? Review of Financial Studies. 2012; 25 (10): 3037-76. <https://doi.org/10.1093/rfs/hhs078>.

*Original manuscript in PDF format:*

14. Nakamura E, Steinsson J, et al. Crises and recoveries in an empirical model of consumption disasters. American Economic Journal: Macroeconomics. 2013; 5(3): 35-74. https://doi.org/10.1257/mac.5.3.35.

*Revised manuscript in WORD format:*

14. Nakamura E, Steinsson J, Barro RJ, Ursúa JF. Crises and recoveries in an empirical model of consumption disasters. American Economic Journal: Macroeconomics. 2013; 5(3): 35-74. https://doi.org/10.1257/mac.5.3.35.

*Original manuscript in PDF format:*

15. Barro RJ, Jin T. Rare events and long-run risks. Review of economic dynamics. 2021; 39: 1-25. https://doi.org/10.1016/j.red.2020.08.002.

*Revised manuscript in WORD format:*

15. Barro RJ, Jin T. Rare events and long-run risks. Review of economic dynamics. 2021; 39: 1-25. https://doi.org/10.1016/j.red.2020.08.002. PMID: 32843834

*Original manuscript in PDF format:*

19. Cox JC, Ingersoll JE, Jr, Ross SA. A theory of the term structure of interest rates. Econometrica. 1985; 53: 385-408. https://doi.org/10.1142/9789812701022_0005.

*Revised manuscript in WORD format:*

19. Cox JC, Ingersoll Jr JE, Ross SA. A theory of the term structure of interest rates. Econometrica. 1985; 53: 385-408. https://doi.org/10.1142/9789812701022_0005.

*Original manuscript in PDF format:*

22. Hall, RE. Intertemporal substitution in consumption. Journal of Political Economy. 1988; 96: 339-357.https://doi.org/10.1111/joes.12142.

*Revised manuscript in WORD format:*

22. Hall RE. Intertemporal substitution in consumption. Journal of Political Economy. 1988; 96: 339-357. https://doi.org/10.1111/joes.12142.

**Point 10:** Proofreading is also required.

**Response 10:** Thank you very much! As shown in Table 5, we proofread the original manuscript.

**Table 5. Proofreading the original manuscript**

| *Original manuscript in PDF format* | | *Revised manuscript in WORD format* | |
| --- | --- | --- | --- |
| Line(s) | Before proofreading | Line(s) | After proofreading |
| Manuscript title | Can an asset pricing model containing both good and bad events with time-varying probability predict the government bill rate volatility? | 3 | Economic events and the volatility of government bill rates |
| 2-5 | Mehra and Prescott [1] used the historical stock data of the United States to conduct empirical research and found that the average annual stock return rates and risk-free rates in the United States from 1889 to 1978 were about 6.98% and 0.8% respectively; that is, the average equity premium in the United States was as high as 6.18%. | 71-74 | Mehra and Prescott [1] find that the average annual stock return and risk-free rate in the United States from 1889 to 1978 were about 6.98% and 0.8%, respectively; that is, the average equity premium in the United States was as high as 6.18%. |
| 5-6 | The traditional | 74 | The early |
| 6 | model | 75 | models |
| 7 | a reasonable risk aversion coefficient | 75-76 | a rational risk aversion coefficient |
| 7-8 | , which was called | 76 | called |
| 9 | account for the equity premium puzzle of Mehra and Prescott. | 77-78 | account for the puzzle. |
| 10 | quantified rare economic disasters and reported | 78-79 | define rare economic disasters and report |
| 11 | defined a | 79-80 | defines a |
| 12 | defined | 81 | define |
| 14-20 | Barro and Ursúa [4] defined a rare economic disaster as a peak-to-trough percent fall in consumption (or real per capita GDP) by at least 10%; the statistical results obtained from consumption data (1870-2006, 22 countries) indicated that, on average, a rare economic disaster reached its trough after 3.6 years, with a peak-to-trough fall in consumption averaging about 21.8% and an average probability of 3.55% per year; the statistical results obtained from GDP data (1870-2006, 35 countries) indicated that, on average, a rare economic disaster reached its trough after 3.5 years, with a peak-to-trough fall in GDP averaging about 20.8% and an average probability of 3.68% per year. | 81-82 | Barro and Ursúa [4] define a rare economic disaster as a peak-to-trough percent fall in consumption (or real per capita GDP) by at least 10%. |
| 21 | (e.g., [3–10]) | 88-89 | [3-10] |
| 22 | the Rietz-Barro model which has | 89-90 | the Rietz-Barro model, which has |
| 22 | a very | 90 | an |
| 24 | , etc. | 91 | . |
| 25-27 | Gourio [13] points out that less attention has been paid to the growth component of consumption than to rare disasters | 94-95 | Gourio [13] points out that the growth component of consumption has received insufficient attention |
| 28-29 | the model of Nakamura et al. [14] | 96 | Nakamura et al. [14] |
| 29 | allows for | 97 | allow for |
| 30 | expanded | 98 | expand |
| 31 | argued | 98 | argue |
| 36 | (e.g., [4,7,15,16]) | 103 | [4,7,15,16] |
| 39 | and | 105 | , and |
| 39 | respectively, while | 106 | respectively. In comparison, |
| 40 | and | 107 | , and |
| 44 | rare disasters, but | 111 | rare disasters but |
| 48 | (e.g. 3%) | 115 | (e.g., 3%) |
| 49-51 | Bad events include rare economic disasters, with the former having a higher average probability but a smaller average scale than the latter. | 116-117 | Bad events include rare economic disasters, and good events are the opposite of bad events. |
| 57 | than consumption itself | 123 | than consumption |
| 60 | falls by 4.82% while | 126 | fell by 4.82%, while |
| 61 | consumption falls | 126 | consumption fell |
| 61-63 | During COVID-19 which has not yet ended, the consumption growth rate has fallen by 6.54% while consumption has fallen by only 62  4.12%. | 126-128 | During COVID-19, which has not yet ended, the consumption growth rate has fallen by 6.54%, while consumption has fallen by only 62  4.12%. |
| 63 | an obvious symmetry | 128 | an apparent symmetry |
| 64 | curves; it means that good events | 129 | curves. Thus, good events |
| 66 | the following volatility characteristics of U.S. consumption growth rates | 131-132 | the following characteristics of the U.S. consumption growth rate |
| 67 | (1) | 132 | (i) |
| 67 | (2) | 132 | (ii) |
| 68 | an average | 133 | the average |
| 69 | (3) | 134 | (iii) |
| 70 | an average | 135 | the average |
| 71-72 | Because of the asymmetry in consumption curves | 135-136 | Since there is no mean for consumption |
| 73 | in consumption growth rate curves | 138 | in the consumption curve |
| 74-77 | Thus, in the Rietz-Barro model, the drift of consumption growth rates is usually a constant greater than the long-term consumption growth rate; the excess is used to reflect the impact of good events (e.g., institutional reform, management innovation, technological progress, etc.). | 141-144 | As a result, in the Rietz-Barro model, the drift of consumption growth rates is typically a fixed amount higher than the long-term consumption growth rate; the excess is utilized to indicate the influence of good events (e.g., institutional reform, management innovation, technological progress). |
| 80 | ; a | 150 | . A |
| 84-87 | First, both good and bad events are modeled instead of only disasters being modeled. Second, economic events, rather than having an effect that lasts only a period, have an ongoing effect. Third, more economic events are counted by our definition of economic events, not fewer ones. | 153-156 | First, model both good and bad events, not just bad events (e.g., rare economic disasters). Second, the event’s impact lasts for multiple periods rather than one period. Third, model non-rare economic events. |
| 88 | In this paper, we construct | 160 | We construct |
| 90-91 | A good event and a bad event are modeled as a positive scale jump and a negative scale jump, respectively; we | 162-163 | A good event and a bad one are modeled as a positive scale jump and a negative one, respectively. We |
| 96 | that set | 168 | that sets |
| 98-99 | time-varying event | 170 | time-varying |
| 104-108 | In addition, the solution for the model implies that the inclusion of good economic events with time-varying probability in the Rietz-Barro disaster model will further enhance the time-varying characteristics of asset returns, but this has no obvious influence on average equity premium than with static probability. | 179-182 | Moreover, the solution for our model implies that including good events with time-varying probability in the Rietz-Barro disaster model can further enhance the time-varying characteristics of asset returns, but it has no significant effect on the average equity premium. |
| 110-111 | it can match | 184 | it can generate both |
| 111 | as well as | 185 | and |
| 113 | Our model | 187 | Moreover, our model |
| 114-115 | match equity premium and the high equity return volatility; that is, the Sharpe ratio can also be well matched. | 187-188 | match the equity premium, the equity return volatility, and the Sharpe ratio. |
| 123-130 | can the continuous-time models price assets better than the discrete-time model? The continuous-time model of Wachter [7] and Barro and Jin [20] shows that people will increase precautionary savings to solve disaster risks. Since relative risk aversion is usually greater than one, we infer that in the continuous-time model, the effects of good and bad events on people's precautionary savings are opposite but asymmetric; however, in our discrete-time model, their effects are opposite and symmetric. Therefore, it is necessary to reexamine this study under a continuous-time model in future research. | 196-200 | is it appropriate to assume the size of events to be constant? Both Wachter [7] and Barro and Jin [20] assume that the disaster size follows some probability density function. Therefore, it is necessary to endow the event size with time-varying characteristics in future research. |
| 133 | There are few kinds of literature exploring | 208-209 | Few kinds of literature explore |
| 135-136 | Several recent papers focusing on recoveries after disasters have some similarities with this paper. In this paper, good events include recoveries after disasters. | 210-213 | This paper and several recent papers on disaster recoveries share certain parallels. Good events in this article include recoveries after disasters. |
| 139 | disasters and do | 215 | disasters. They do |
| 139-140 | curve that is not related | 216 | unrelated |
| 141-143 | The rest of the paper proceeds as follows. Section 2 describes the data. Section 3 builds the model. Section 4 shows closed-form solutions of the model. Section 5 is the simulation. |  |  |
| 144 | A description of U.S. consumption growth rate data | 219-220 | U.S. consumption growth rates and economic events |
| 145 | In U.S. consumption growth rate data (1870-2020), we find a total of 44 | 221-222 | U.S. consumption growth rate data (1870-2020) shows 44 |
| 146-147 | Fig 2 shows the size distribution of the rises and declines in the data. | 222-223 | Fig 2 shows the size distribution of the rises and declines. |
| 147-148 | In general, the number of larger sizes of rises (or declines) is less than the number of smaller sizes of rises (or declines). | 224-225 | Generally, the number of larger rises (or declines) is less than the number of smaller rises (or declines). |
| 151 | Barro and Ursúa [4], but as shown in Fig 3, it's not | 227-228 | Barro and Ursúa [4]. However, as shown in Fig 3, it is |
| 152 | good fit to | 228 | good fit for |
| 154 | declines in the data | 231 | declines |
| 156-157 | **The distribution of transformed sizes of** | 235 | **The transformed size distribution of** |
| 158 | $\leq$ | 240 | $<$ |
| 159 | $\leq$ | 241 | $<$ |
| 159-160 | Fig 5 shows descriptive statistics of events with different event thresholds. | 241-242 | Fig 5 shows the correspondence between the thresholds ($\tau^{r}$ and $\tau^{d}$) and the characteristics of economic events. |
| 162-164 | The average cumulative effect of good events increases in the threshold, while the average cumulative effect of bad events decreases in the threshold. | 245-246 | The average cumulative effect of both good and bad events increases in the threshold. |
| 165-166 | good events, while it | 248 | good events. In comparison, it |
| 169 | in the average probability | 251 | in the probability |
| 172 | $\delta$ | 257 | $1-\delta$ |
| 175 | Epstein-Zin utility | 260 | recursive utility |
| 175-176 | This feature of Epstein-Zin utility | 261 | recursive utility |
| 176-177 | in future consumption as well as future utility | 261 | to future risks |
| 178 | close to 1, | 264 | close to 1 |
| 183 | that, when $\psi=1.5$, the model | 267 | that when $\psi$ is equal to 1.5, the model |
| 185 | In this paper, we | 270 | We |
| 187 | (1) | 272 | Eq. (1) |
| 189 | (1) | 719 | Eq. (1) |
| 193 | (2) | 278 | Eq. (2) |
| 193 | (1) | 279 | Eq. (1) |
| 195 | (2) | 280 | Eq. (2) |
| 199 | log consumption growth rates | 281 | the log consumption growth rate |
| 201 | i.e. economic events | 288 | i.e., economic events |
| 202 | scale | 289 | size |
| 202 | (4) | 289 | Eq. (4) |
| 202 | (3) | 289 | Eq. (3) |
| 203-204 | rates; therefore, our model incorporates some elements similar to the long-run risk model | 290-292 | rates. So, it can be said that our model is somehow related to the long-run risk model |
| 209 | Eq.(4) | 296 | Eq. (4) |
| 209 | (3), (4) and (5) | 297 | Eqs. (3), (4), and (5) |
| 211 | (5) | 298 | Eq. (5) |
| 218 | (2) | 306 | Eq. (2) |
| 219 | To obtain the closed-form solution of the log stochastic discount factor, assumption 1 is given first. | 309-310 | First, assumption 1 is made to get the closed-form solution for the log stochastic discount factor. |
| 227 | (5) | 728 | Eq. (5) |
| 232 | (9) | 326 | Eq. (B1) |
| 234-235 | Therefore, to obtain the closed-form solution of $m_{t+1}$, other solutions for unknown variables $v_{t}$ and $\chi_{t}^{2}$ and unknown coefficients $\omega_{1}$ and $\omega_{2}$ are required. |  |  |
| 236 | (2) | 331 | Eq. (2) |
| 237 | (2) | 731 | Eq. (2) |
| 239 | (11) | 733 | Eq. (10) |
| 239 | (12) | 733 | Eq. (11) |
| 251 | (12) | 739 | Eq. (11) |
| 255 | (13), (14) and (15) | 748 | Eqs. (D1), (D2), and (D3) |
| 256 | (16) and (17) | 749 | Eqs. (D4) and (D5) |
| 257 | (16) and (17) | 750 | Eqs. (D4) and (D5) |
| 258 | (16) and (17) | 751 | Eqs. (D4) and (D5) |
| 266 | To obtain the closed-form solution of the log risk-free rate, assumption 4 is given first. | 371 | Assumption 4 is given first to get the closed-form solution of the log risk-free rate. |
| 272 | (21) | 382 | Eq. (15) |
| 275-277 | Since we assume that $N_{i}$ follows a Bernoulli distribution rather than a Poisson distribution, precautionary savings are unaffected by economic events in our discrete-time model. |  |  |
| 277 | (21) | 387 | Eq. (15) |
| 278 | (21) | 388 | Eq. (15) |
| 279 | (22) | 389 | Eq. (16) |
| 285 | line. The | 394 | line—the |
| 287 | the risk-free rate volatility. | 396 | risk-free rate volatility. |
| 291 | We follow Colacito and Croce [27] and assume | 406 | We refer to Colacito and Croce [27] to assume |
| 292-293 | (23) | 408 | (17) |
| 295-296 | (24) | 412 | (18) |
| 296 | (24) | 413 | Eq. (18) |
| 297 | assumption 5 and assumption 6 | 415 | assumptions 5 and 6 |
| 306-307 | assumption 5 and assumption 6 | 754-755 | assumptions 5 and 6 |
| 308 | (29) and (30) | 761 | Eqs. (E2) and (E3) |
| 308 | (31) and (32) | 761 | Eqs. (E4) and (E5) |
| 309 | (31) and (32) | 762 | Eqs. (E4) and (E5) |
| 310 | (31) and (32) | 763 | Eqs. (E4) and (E5) |
| 311 | Eq.(28) | 764 | Eq. (E1) |
| 312-314 | a fixed point problem, evaluating the expanded state vector at its unconditional mean $\left( E\left( pc_{t} \right) \right)$. When $\delta=0.988$, the steady state value | 438-439 | a fixed point problem. When $\delta$ is equal to 0.988, the steady-state value |
| 317 | (34) indicates that the expected equity return is affected by both good and bad events. | 447-448 | Eq. (23) indicates that both good and bad events affect the expected equity return. |
| 318 | is increasing | 449 | increases |
| 324-325 | a certain value | 455 | a specific value |
| 326 | Simulation | 463 | Calibration and simulation |
| 327-336 | Barro and Ursúa [4] define the rare economic disaster and argue that the inclusion of expansions between an event threshold of 10% and an event threshold of 15% leaves out many events but has only moderate implications for explaining asset returns; that is, the threshold size is non-critical. A higher threshold means that fewer economic events are counted. To build an asset pricing model that contains as many events as possible, we define a bad (or good) event as a peak-to-trough absolute decline (or a trough-to-peak absolute rise) in consumption growth rates by a low threshold. In our model, is the threshold size still non-critical? To answer this question, in the simulation, we discuss two cases: (i) a very low event threshold (τ^r=τ^d=0) and (ii) a slightly higher event threshold (τ^r=τ^d=3%). |  |  |
| 337 | Parameters | 472 | Calibration |
| 338-355 | In our model, time is measured in years and parameter values are given accordingly.  First, the common parameters are given as follows: $\delta=0.988$, $\gamma=6$, $\phi=0.92$, $\Phi=3$ and $\sigma_{d}=0.15$. The average real return on the 3-month Treasury bill in postwar U.S. data is used as the basis for setting the rate of time preference $\left( \delta\right)$ [7]. The relative risk aversion is set equal to 6 to follow the models of allowing for faster growth following a disaster [13-15,30]. The mean reversion parameter, $\phi$, is calibrated to be consistent with the autocorrelation for the price-dividend ratio in postwar U.S. data [7]. The leverage, $\Phi$, is set equal to 3; this is a reasonable value by the standards of available literature [7,16,29,31]. The dividend-specific volatility, $\sigma_{d}$, is set equal to 0.15 [27].  Second, we discuss event parameters ($\theta_{i}$, $a_{i}$ and $b_{i}$). Table 1 shows descriptive statistics of good and bad events when $\tau^{r}=\tau^{d}=0$ and $\tau^{r}=\tau^{d}=3\%$. In our model, the parameter $\theta_{i}$ determines the average probability of events when $\phi$ is fixed; the larger $\theta_{i}$, the higher the average probability. When the average probability of events is determined, $\theta_{i}$ can be calculated based on (6). The parameter $a_{i}$ determines the duration of events; the larger $a_{i}$, the longer the duration. The parameter $b_{i}$ determines the initial effect of events. When the average duration and cumulative effect of events are known, $a_{i}$ and $b_{i}$ can be determined by fitting. The fitting curves are shown in Fig 11. Event parameters for the simulation are shown in Table 2. | 473-497 | Our model measures time in years, and parameter values are given accordingly. First, we calibrate parameters independent of economic events ($\delta$, $\gamma$, $\phi$, $\Phi$, and $\sigma_{d}$). The rate of time preference ($1-\delta$) is equal to 0.012 to match the average real return on the 3-month Treasury bill in postwar U.S. data [7]. The relative risk aversion ($\gamma$) is equal to 6 to follow the models allowing for faster growth following a disaster [13-15,30]. The mean reversion parameter ($\phi)$ is set to 0.92 to be consistent with the autocorrelation for the price-dividend ratio in postwar U.S. data [7]. The leverage ($\Phi)$ is set to 3, which is a reasonable value by the standards of available literature [7,16,29,31]. The dividend-specific volatility ($\sigma_{d}$) is set to 0.15 [27].  Second, we discuss parameters related to economic events ($\theta_{i}$, $a_{i}$, and $b_{i}$). From Eq. (6), the parameter $\theta_{i}$ determines the event probability when $\phi$ is fixed; the larger $\theta_{i}$, the higher the probability. We can calculate $\theta_{i}$ if the event probability and the parameter $\phi$ are known. The parameter $a_{i}$ measures the duration of an economic event, and the parameter $b_{i}$ represents the initial effect of an economic event. If the average duration and cumulative effect of economic events are known, $a_{i}$ and $b_{i}$ can be determined by fitting. We give specific thresholds ($\tau^{r}$ and $\tau^{d}$) to obtain the average duration, probability, and average cumulative effect of economic events. Barro and Ursúa [4] argue that raising the threshold from 10% to 15% when defining rare economic disasters will exclude many economic events but has only moderate implications for explaining asset returns. We consider two cases with thresholds ($\tau^{r}$ and $\tau^{d}$) equal to 0 and 3% to test the applicability of Barro and Ursúa's above statement to this paper. Table 1 shows descriptive statistics of good and bad events when $\tau^{r}=\tau^{d}=0$ and $\tau^{r}=\tau^{d}=3\%$. Fig 11 shows the fitting curves drawn according to the average duration and cumulative effect provided in Table 1. Table 2 presents parameters $a_{i}$ and $b_{i}$ back-calculated from the fitted curves in Fig 11. |
| 355-356 | Average probability (%) | 514-515 | Probability (%) |
| 357-363 | To facilitate discussion for the simulation results, we rewrite (3) as  $\Delta c_{t+1}=\mu+\sigma\varepsilon_{t+1}+\iota_{1}\eta_{1,t+1}+\iota_{2}\eta_{2,t+1},$ (36)  where $\iota_{1}\in\left\{ 0,1 \right\}$ and $\iota_{2}\in\left\{ 0,1 \right\}$. In different specifications of the model, $\iota_{1}$ and $\iota_{2}$ have different values. The model is simulated at an annual frequency for 50,000 years. If the event probability $(\lambda_{i,t})$ is negative or greater than one, we set $\lambda_{i,t}=\lambda_{i,t-1}$. Simulation results with $\tau^{r}=\tau^{d}=0$ are shown in Table 3, and simulation results with $\tau^{r}=\tau^{d}=3\%$ are shown in Table 4. Both Tables 3 and 4 consist of three parts: (1) specific parameters, (2) assumption verifications, and (3) moments. | 524-539 | In this subsection, we compare the simulation results of our model with those of other models to characterize our model. All models are simulated at an annual frequency for 50,000 years.  Table 3 shows the simulation results with $\tau^{r}=\tau^{d}=0$, and it mainly consists of three parts: (i) other parameters, (ii) assumption verifications, and (iii) moments. The values of other parameters ($\mu$, $\sigma$, and $\sigma_{\lambda}$) are determined by matching the moments from U.S. data. Specifically, the value of the parameter $\mu$ is determined by matching the average consumption growth rate, and the values of parameters $\sigma$ and $\sigma_{\lambda}$ are determined by matching the consumption growth volatility. Before giving the moments of the models, we verify assumption 2 whose measurement is $\Gamma_{i}$, assumption 4 whose measurement is $\Lambda_{i}$, and assumption 6 whose measurement is $\Upsilon_{i}$, where $i\in\{1,2\}$. Finally, we report moments from both U.S. data and the models. The U.S. Data from Barro and Ursúa [4]. Because we assume that the probability of default on the short-term government bill in the United States is equal to zero, the short-term government bill rate is equal to the risk-free rate. In Table 3, $No$ represents a model that does not include economic events, $Only\_bad$ represents a model that includes only bad events, and $Both$ represents a model that includes both good and bad events. |
| 373-374 | Moments of the model | 548 | Moments |
| 373-374 | Model | 548-549 | Models |
| 373-374 | Moments of the model | 597 | Moments |
| 373-374 | Model | 597-598 | Models |
| 364-457 | In part of specific parameters, $\iota_{1}$ and $\iota_{2}$ jointly determine the modeling characteristics of the model for economic events. Both good and bad events are modeled when $\iota_{1}=1$ and $\iota_{2}=1$; only bad events are modeled when $\iota_{1}=0$ and$\iota_{2}=1$; only good events are modeled when $\iota_{1}=1$ and$\iota_{2}=0$; there are no economic events in the model when $\iota_{1}=0$ and$\iota_{2}=0$. Other specific parameters, $\mu$, $\sigma$, and $\sigma_{\lambda}$, are determined by matching the moments in U.S. data. In our consumption-based asset pricing model, $\mu$, the constant term of the log consumption growth rate process, is used to match the average consumption growth rate in normal times; $\sigma$and$\sigma_{\lambda}$, for the consumption shock and the volatility parameter of event probability respectively, are used to match the consumption growth rate volatility. When economic events are contained in the model, $\sigma_{\lambda}$ indicates that the event probability is static, and $\sigma_{\lambda}>0$ means that it is time-varying. In part of assumption verifications, we verify assumption 2 whose measurement is $\Gamma_{i}$, assumption 4 whose measurement is $\Lambda_{i}$, and assumption 6 whose measurement is $\Upsilon_{i}$, where $i\in\{1,2\}$. In part of moments, we report moments from both U.S. data and our model. The U.S. Data from Barro and Ursúa [4]. Wachter [7] shows that the risk-free rate volatility is an upper bound on the government bill rate volatility and sets the probability of default given disaster equal to 0.4 by referring to Barro and Ursúa [4]. We assume that the probability of default on short-term government liabilities in the United States is equal to zero, so the short-term government bill rate is the risk-free rate.  Simulation results with a very low event threshold  Table 3 shows simulation results with $\tau^{r}=\tau^{d}=0$ and contains five specifications. For each specification, assumption 2, assumption 4, and assumption 6 are true; the maximum error is less than 1.5%. We use the specifications (1), in which economic events are not modeled ($\iota_{1}=\iota_{2}=0$), as a benchmark. In specifications (1), to match the average consumption growth rate and the standard deviation of consumption growth rates, $\mu=0.0185$ and $\sigma=0.036$ are required; except for the standard deviation of government bill rates, other moments can be well-matched with the data; the standard deviation of government bill rates predicted by the model (0.00%) is substantially lower than that observed in U.S. data (4.82%).  In specifications (2) and (3), we add the modeling of economic events with static probability (i.e. $\sigma_{\lambda}=0$). In specification (2), the model contains only bad events (i.e. $\iota_{1}=0$ and $\iota_{2}=1$); in specification (3), the model contains both good and bad events (i.e. $\iota_{1}=1$ and $\iota_{2}=1$). We first adjust parameter $\sigma$ in the specification (3) to match the standard deviation of consumption growth rates, requiring $\sigma$ to be equal to 1.9%. To facilitate comparison, we assume that $\sigma$ is also equal to 1.9% in the specification (2). To match the average consumption growth rate, $\mu=3.47\%$ is required in specifications (2) and $\mu=1.85\%$ is required in specifications (3). The standard deviation of government bill rates in specifications (2) is 0.33%, while it is 0.46% in specifications (3). As (22) implies, this result shows that the modeling of both good and bad events can improve the standard deviation of government bill rates compared to the modeling of only bad events. Nevertheless, the standard deviation of government bill rates in specifications (3) is still much lower than that observed in U.S. data (4.82%) and the standard deviation of consumption growth rates at this time (3.60%). That is, the model containing economic events with static probability does not explain the volatility puzzle focused on in this paper. The standard deviation of consumption growth rates in specifications (3) is 3.60%, while it is 2.88% in specifications (2); the standard deviation of equity return rates in specifications (3) is 18.49%, while it is 17.30% in specifications (2).  In specifications (4) and (5), we examine the modeling of economic events with time-varying probability (i.e. $\sigma_{\lambda}=0$). In specification (4), the model contains only bad events (i.e. $\iota_{1}=0$ and $\iota_{2}=1$); in specification (5), the model contains both good and bad events (i.e. $\iota_{1}=1$ and $\iota_{2}=1$). In our model, the consumption growth rate volatility is an increasing function of both $\sigma$ and $\sigma_{\lambda}$ (see (3)); the government bill rate volatility is an increasing function of $\sigma_{\lambda}$, independent of $\sigma$ (see (22)). Thus, to match the consumption growth rate volatility, $1.9\%>\sigma\geq0$ is required in the specification (5); to simultaneously match the consumption growth rate volatility with the high government bill rate volatility, we assume that the value of $\sigma$ is very small in order to maximize the value of $\sigma_{\lambda}$. Based on the same logic, we first adjust parameter $\sigma_{\lambda}$ in the specification (5) to match the standard deviation of consumption growth rates; so that requires $\sigma_{\lambda}$ to be equal to 0.38 when we set $\sigma=0.5\%$. To facilitate comparison, we assume $\sigma_{\lambda}$ equal to 0.38 and then take $\sigma$ equal to 0.5% in the specification (4). The standard deviation of government bill rates in specifications (4) is 3.19%, while it is 4.50% in specifications (5). Again, this result shows that, compared to the modeling of only bad events, the modeling of both good and bad events can significantly increase the government bill rate volatility. When the event threshold is equal to zero, the model containing both good and bad events with high time-varying probability is able to explain the volatility puzzle focused on in this paper. The standard deviation of consumption growth rates in specifications (5) is 3.60%, while it is 2.57% in specifications (2); the standard deviation of equity return rates in specifications (5) is 18.47%, while it is 16.87% in specifications (2).  In general, our model can also quantitatively match equity premium and the high equity return volatility; this means that the Sharpe ratio can also be well matched. A downside is that the government bill rate predicted by our model is higher than that observed in the data, because, in our discrete-time model, neither good events nor bad events will affect the preventive saving of residents. If our model is modified to a continuous-time model, this downside will disappear but the model will become extremely complex.  Simulation results with a slightly higher event threshold  Simulation results with $\iota_{1}=\iota_{2}=3\%$ demonstrate the model's ability to explain asset returns at a higher event threshold, as illustrated in Table 4. For the sake of exposition, we retain the same presentation used in Table 3. Similar conclusions can be drawn from Table 4 as from Table 3. By comparing the results in Tables 3 and 4, however, we obtain three new findings. Firstly, regardless of whether the event probability is static or time-varying, economic events can bring in higher consumption growth rate volatility at a higher event threshold. Secondly, economic events with static probability bring higher government bill rate volatility at a higher event threshold; however, economic events with time-varying probability bring lower government bill rate volatility at a higher event threshold. Thirdly, to explain the volatility puzzle focused on in this paper, in addition to high time-varying probability and the modeling of both good and bad events, it is also necessary to set an event threshold close to zero. Together, these three findings suggest that, based on our definition of economic events, minor but numerous events with high time-varying probabilities are very important for understanding the government bill rate volatility. | 550-596 | Table 3 provides five specifications. For each specification, assumptions 2, 4, and 6 all hold; the maximum error is less than 1.5%. Specification 1 is our benchmark, which demonstrates the performance of a model that does not include economic events. In specification (1), $\mu$ and $\sigma$ are set to 1.85% and 3.6% to match the average growth rate of consumption and the standard deviation of consumption growth. Specification 1 shows that, with the exception of the standard deviation of government bill rates, the other moments match the data well.  Specifications (2) and (3) feature the modeling of economic events with static probability (i.e., $\sigma_{\lambda}=0$ and $\lambda_{i}>0$). We first set the parameter $\sigma$ in specification 3 equal to 1.9% to match the standard deviation of consumption growth. Then, we also set the parameter $\sigma$ in specification 2 to 1.9%. Therefore, comparing specifications (2) and (3) can reflect the impact of adding the modeling of good events with static probability on the standard deviation of asset returns. The standard deviation of government bill rates in specifications (2) is 0.33%, while it is 0.46% in specifications (3). Nevertheless, the standard deviation of government bill rates in specifications (3) is still much lower than that of consumption growth (3.60%). These results suggest that an asset pricing model that includes both good and bad events with static probability cannot explain the volatility puzzle that this paper focuses on.  Specifications (4) and (5) examine the modeling of economic events with time-varying probability (i.e., $\sigma_{\lambda}>0$ and $\lambda_{i}>0$). Specification (5) presents the performance of our model containing both good and bad events with time-varying probability, while specification (4) appears the performance of a model that includes only bad events with time-varying probability. From Eq. (16), if the probability of an economic event is time-varying, the government bill rate volatility is an increasing function of $\sigma_{\lambda}$. Also, from Eq. (3), if the probability of an economic event is time-varying, the consumption growth volatility is an increasing function of both $\sigma$ and $\sigma_{\lambda}$. Therefore, in specifications (4) and (5), the larger the parameter $\sigma_{\lambda}$ and the smaller the parameter $\sigma$, the more favorable it is to generate the government bill rate volatility higher than the consumption growth volatility. First, we determine the values of parameters $\sigma$ and $\sigma_{\lambda}$ in specification (5). If we assume that the value of parameter $\sigma$ in specification 5 is equal to 0.5% (a very small value), the value of parameter $\sigma_{\lambda}$ (0.38) in specification (5) can be inversely calculated by matching the consumption growth volatility of 3.6%. Second, for contrast between specifications (4) and (5), we set the values of parameters $\sigma$ and $\sigma_{\lambda}$ in specification (4) to be the same as those in specification (5). The standard deviation of consumption growth in specifications (4) is 2.57%, while it is 3.60% in specifications (5). The standard deviation of government bill rates in specifications (4) is 3.19%, while it is 4.50% in specifications (5). These results show that adding the modeling of good events with time-varying probability can significantly increase the government bill rate volatility and the consumption growth volatility. While both specifications (4) and (5) present the government bill rate volatility higher than consumption growth volatility, it is clear that the moments in specification (5) match that from the U.S. data better. Overall, each of the specifications in Table 3 quantitatively matches the equity premium, the high equity return volatility, and the Sharpe ratio.  Table 4 demonstrates the simulation results with $\tau^{r}=\tau^{d}=3\%$. We obtain three new findings by comparing the results in Tables 3 and 4. Firstly, whether the event probability is static or time-varying, economic events can bring in the higher consumption growth volatility at a higher event threshold. Secondly, economic events with static probability bring the higher government bill rate volatility at a higher event threshold; however, economic events with time-varying probability bring the lower government bill rate volatility at a higher event threshold. Thirdly, a near-zero event threshold is a key to our theoretical model's ability to explain the volatility puzzle that this paper focuses on. |
| 459-481 | In this study, we set out to address the following puzzle: the government bill rate volatility far exceeds the consumption growth rate volatility. We did so by extending the Rietz-Barro model's definition of rare economic disasters and proposing an asset pricing model that contains both good and bad events. Compared with the Rietz-Barro model, our model contains three modifications: (1) both good and bad events are modeled instead of only disasters being modeled, (2) economic events, rather than having an effect that lasts only a period, have an ongoing effect, and (3) more economic events are counted by our definition of economic events, not fewer ones. Our model is solved in closed form. Simulation results suggest that our model can quantitatively match equity premium and the high equity return volatility; more importantly, it can predict high government bill rate volatility. In this paper, three elements are needed to bring the government bill rate volatility higher than the consumption growth rate volatility: (1) the modeling of both good and bad events, (2) the high time-varying probability, and (3) an event threshold close to zero. As described by Wachter [7], compared with static probability, time-varying probability drives higher asset return volatility. In consumption-based asset pricing models, the modeling of both good and bad events implies that the consumption growth rate is in the tail with some probability not just in the left tail. Setting the event threshold close to zero helps maximize the volatility of event probability, and the government bill rate volatility is an increasing function of the volatility of event probability. This paper argues that the inclusion of good economic events with time-varying probability in the Rietz-Barro disaster model is an important complement to understanding asset return volatility, especially the government bill rate volatility. | 705-717 | In many countries (especially G7), there is a volatility puzzle: the government bill rate volatility far exceeds the consumption growth rate volatility [4,7,15,16]. To address the puzzle, we extend the traditional definition of rare economic disasters and propose an asset pricing model that contains both good and bad events with time-varying probability. Compared with traditional disaster models, our model contains three modifications: (i) model good and bad events, not just bad ones (e.g., rare economic disasters); (ii) the event’s impact lasts for multiple periods rather than one period; (iii) model non-rare economic events. In our model, a near-zero event threshold is key to bringing the government bill rate volatility higher than the consumption growth volatility. Our modeling of both good and bad events implies that the consumption growth rate is in the tail with some probability, not just in the left tail. We argue that the inclusion of good events with time-varying probability in the traditional rare disaster model is an essential complement to understanding asset return volatility, especially the government bill rate volatility. |

**Response to Reviewer 2's Comments**

**Point 1:** Check this introduction, p. 4, lines 95 and 110, there are capital letters missing.

**Response 1:** Thank you very much for pointing out these errors. As shown in Table 6, inspired by you, we have revised all similar errors in the original manuscript.

**Table 6. Correct initial letter errors in the original manuscript.**

| *Original manuscript in PDF format* | | *Revised manuscript in WORD format* | |
| --- | --- | --- | --- |
| Line 95 | our model | Line 167 | Our model |
| Line 110 | our model | Line 184 | Our model |

**Point 2:** Please see if Cochrane (2005) provides a relevant discussion on the equity premium puzzle.

**Response 2:** Yes. Chapter 21 (pp. 455-486) of Cochrane (2005) documents a discussion of the equity premium puzzle. We read this chapter carefully. Although our research focuses on another asset pricing puzzle, we learn a lot from this reading. Moreover, this reading prompts us to think deeply about the shortcomings of our theoretical framework and the question of how to improve our theoretical framework in the future.
